# Diagnostic Performance of Chimeric Recombinant Antigens of *Trypanosoma cruzi* for Identifying Chagas Disease in Samples From Rio Grande do Sul

**DOI:** 10.1111/tmi.70019

**Published:** 2025-08-07

**Authors:** Ângelo Antônio Oliveira Silva, Ítalo Ferreira de Leon, Felipe Silva Santos de Jesus, Daniel Dias Sampaio, André Luis Bartz Voigt, Natália Berne Pinheiro, Nathieli Bianchin Bottari, Leda Margarita Castaño‐Barrios, Paola Alejandra Fiorani Celedon, Nilson Ivo Tonin Zanchin, Fabricio Klerynton Marchini, Maria Elisabeth Aires Berne, Fred Luciano Neves Santos

**Affiliations:** ^1^ Advanced Health Public Laboratory Goncalo Moniz Institute, Oswaldo Cruz Foundation Salvador Bahia Brazil; ^2^ Interdisciplinary Research Group in Biotechnology and Epidemiology of Infectious Diseases (GRUPIBE), Goncalo Moniz Institute, Oswaldo Cruz Foundation Salvador Bahia Brazil; ^3^ Laboratory for Parasitology, Postgraduate Program in Microbiology and Parasitology Federal University of Pelotas Pelotas Rio Grande do Sul Brazil; ^4^ Laboratory for Applied Science and Technology in Health Carlos Chagas Institute, Oswaldo Cruz Foundation Curitiba Paraná Brazil; ^5^ Structural Biology and Protein Engineering Laboratory Carlos Chagas Institute, Oswaldo Cruz Foundation Curitiba Paraná Brazil; ^6^ Integrated Translational Program in Chagas Disease From Fiocruz‐Fio‐Chagas, Vice Presidency of Research and Biological Collections, Oswaldo Cruz Fountation Rio de Janeiro Rio de Janeiro Brazil; ^7^ Molecular Biology Institute of Paraná (IBMP) Curitiba Paraná Brazil

**Keywords:** chimeric recombinant antigens, ELISA, serological diagnosis, *Trypanosoma cruzi*

## Abstract

**Background:**

Chagas disease, caused by *Trypanosoma cruzi*, remains a significant public health challenge in Latin America, with diagnostic limitations hindering control efforts.

**Aim:**

Our study aimed to assess the diagnostic performance of four chimeric recombinant *T. cruzi* antigens (IBMP‐8.1, IBMP‐8.2, IBMP‐8.3 and IBMP‐8.4) in a highly endemic region in southern Brazil.

**Methods:**

Serum samples from 333 individuals residing in Canguçu, Rio Grande do Sul, were tested using an in‐house ELISA platform. We assessed the sensitivity, specificity, accuracy, and diagnostic odds ratio (DOR) of individual and combined IBMP antigens through serial and parallel testing strategies.

**Results:**

All antigens exhibited 100% specificity and high accuracy (≥ 93.4%), with IBMP‐8.1 and IBMP‐8.4 showing the best overall performance (sensitivities of 80.0% and 76.7%; DORs of 109,136 and 89,659, respectively). Parallel testing using the combinations IBMP‐8.1 + IBMP‐8.3 and IBMP‐8.1 + IBMP‐8.4 achieved ≥ 95% sensitivity and > 99% accuracy.

**Conclusions:**

These findings support the use of IBMP chimeric antigens, particularly in combination, as reliable tools for Chagas disease diagnosis and surveillance, and highlight the importance of region‐specific validation to ensure diagnostic equity in diverse endemic settings.

## 1 Introduction

Chagas disease (CD), caused by the protozoan *Trypanosoma cruzi*, remains a significant global health concern, affecting an estimated 7 million people worldwide, predominantly in Latin America [1]. The disease is classified as a neglected tropical disease due to its disproportionate burden on low‐income populations and the historical lack of investment in research and treatment options. Despite ongoing efforts to control transmission, CD persists as a major public health challenge, particularly in endemic regions such as Brazil, where seroprevalence varies across different geographical areas [2, 3].

Accurate laboratory diagnosis of CD is essential for patient management and epidemiological surveillance. However, serological detection of anti‐ *T. cruzi* antibodies in chronic infections remains challenging due to the absence of a standardised reference test and the variability in diagnostic accuracy of commercial immunoassays [4, 5]. The sensitivity and specificity of these assays are influenced by the antigenic composition of the tests and the genetic diversity of *T. cruzi* strains circulating in different regions [6]. Given these limitations, alternative diagnostic approaches are needed to improve reliability, particularly in endemic areas where false‐positive and false‐negative results can significantly impact disease control strategies [7].

Recent advancements in recombinant antigen technology have led to the development of chimeric proteins that incorporate multiple immunodominant epitopes from different *T. cruzi* antigens [8, 9]. These chimeric antigens have shown promise in overcoming regional variability and improving diagnostic accuracy [10, 11]. The IBMP chimeric antigen panel [12], which includes IBMP‐8.1, IBMP‐8.2, IBMP‐8.3 and IBMP‐8.4, has been extensively evaluated in different populations and demonstrated high sensitivity and specificity in detecting anti‐ *T. cruzi* antibodies [13, 14, 15, 16]. However, further validation is required to confirm their performance in diverse endemic settings, such as the southernmost region of Brazil.

This study aims to evaluate the diagnostic performance of IBMP chimeric *T. cruzi* antigens in detecting anti‐ *T. cruzi* antibodies in individuals from Canguçu, an endemic municipality in Rio Grande do Sul, a state located in southern Brazil.

## 2 Materials and Methods

### 2.1 Ethical Consideration

This study was approved by the Institutional Review Board (IRB) for Human Research at the Federal University of Pelotas (UFPel), Pelotas, Rio Grande do Sul, Brazil (protocol no. 64461722.7.0000.5317). Written informed consent was obtained from all participants prior to enrolment.

### 2.2 Sampling and Study Design

A cross‐sectional study was conducted to determine the prevalence of *T. cruzi* infection and assess the diagnostic accuracy of IBMP chimeric antigens. The study targeted individuals aged ≥ 18 years who accessed public or private healthcare services, including Basic Health Units (UBS), Emergency Care Units (PA), and private clinics, in the endemic municipality of Canguçu, Rio Grande do Sul, southern Brazil.

Sampling was carried out from December 2022 to May 2024, with participants randomly recruited during routine healthcare visits. Before recruitment, healthcare professionals attended informational sessions to ensure a clear understanding of the study's objectives and methodology.

A sample size of 300 individuals was calculated to ensure sufficient statistical power for estimating *T. cruzi* seroprevalence. Venous blood samples (5 mL) were collected by certified nurses or technicians using standard venipuncture procedures and transported under strict temperature‐controlled conditions to the Human Parasitology Laboratory at the UFPel for serum processing. The sera were then forwarded to the Advanced Public Health Laboratory at the Gonçalo Moniz Institute (Fiocruz‐BA) for serological analysis. Each sample was given a unique identifier code to ensure a blinded analysis.

### 2.3 Laboratory Analysis

Serological testing was conducted using two ELISA‐based assays employing distinct antigen preparations [17, 18]. The Gold ELISA Chagas (Rem Diagnostica, São Paulo, Brazil) utilised both recombinant antigens and purified lysates derived from Brazilian *T. cruzi* epimastigote strains, whereas the Biolisa Chagas Recombinante (Bioclin, Belo Horizonte, Brazil) relied exclusively on recombinant *T. cruzi* antigens. Samples with discordant results between these two assays were subjected to a third confirmatory test: Chagatest ELISA Recombinante v.4.0 (Wiener Lab., Rosario, Argentina). All procedures strictly followed the manufacturers' instructions to ensure accuracy and reliability.

### 2.4 Production of Recombinant Antigens

Chimeric recombinant *T. cruzi* antigens (IBMP‐8.1, IBMP‐8.2, IBMP‐8.3 and IBMP‐8.4) were produced according to established protocols [12]. Briefly, synthetic genes encoding these antigens were synthesised (GenScript, Piscataway, NJ, USA) and subcloned into the pET28a expression vector. *Escherichia coli* BL21‐Star (DE3) cells were used as host for expression of each recombinant antigen using 0.5 M of isopropyl β‐D‐1‐thiogalactopyranoside (IPTG) as expression inductor under optimal conditions to enhance yield and solubility. The recombinant antigens were purified via affinity and ion exchange chromatography, and protein quantification was performed using a fluorometric assay (Qubit 2.0, Invitrogen Technologies, Carlsbad, CA, USA).

### 2.5 In‐House IBMP‐Indirect ELISA

Immunoassays were performed according to established protocols [13]. Transparent high‐binding 96‐well microplates (Greiner Bio‐One, Germany) were coated with chimeric IBMP antigens at a concentration of 12.5 ng/well for IBMP‐8.2 and 25 ng/well for IBMP‐8.1, IBMP‐8.3 and IBMP‐8.4. Antigen coating was performed using a 0.05 M carbonate–bicarbonate buffer (pH 9.6). Microplates were blocked with 50 μL of Well Champion reagent (Kem‐En‐Tec, Denmark) as per the manufacturer's instructions. Serum samples were diluted 1:100 in 0.05 M phosphate‐buffered saline (PBS, pH 7.2), and 50 μL of the diluted samples were added to each well. Microplates were incubated at 37°C for 60 min, followed by washing with PBS containing 0.05% Tween‐20 (PBS‐T, pH 7.4). Horseradish peroxidase (HRP)‐conjugated goat anti‐human IgG (Bio‐Manguinhos, Fiocruz, Brazil), diluted 1:40,000 in PBS‐T, was then added, followed by a 30‐min incubation at 37°C. After a final washing step, 50 μL of TMB substrate (Kem‐En‐Tec Diagnostics A/S, Denmark) was added, and the microplates incubated at room temperature for 10 min in the dark. The enzymatic reaction was stopped with 25 μL of 0.3 M H₂SO₄. Optical density (OD) was measured at 450 nm using a SPECTRAmax 340PC microplate reader (Molecular Devices, San Jose, CA, USA).

### 2.6 Statistical Analysis

Data analysis and visualisation were conducted using Prism v. 9.5.1 (GraphPad, USA). Descriptive statistics were expressed as median and interquartile range (IQR). The Shapiro–Wilk test was used to assess normality. For non‐normally distributed data, the Mann–Whitney signed‐rank test was applied, whereas Student's *t*‐test was used for normally distributed data. All statistical tests were two‐tailed, with a significance threshold set at *p* < 0.05.

Cut‐off values for IBMP‐ELISA assays were determined by analysing five *T. cruzi* ‐positive and five *T. cruzi* ‐negative samples across all microplates. These samples were previously classified as positive or negative based on two independent serological tests, following international guidelines [17, 18]. The cut‐off values were established by maximising the area under the receiver operating characteristic (ROC) curve, optimising the optical density (OD) threshold for discriminating between reactive and nonreactive samples. Results were reported as the reactivity index (RI), calculated as the ratio of the sample OD to the CO OD. Samples with RI values ≥ 1.00 were classified as positive, whereas those within the indeterminate range (RI = 1.00% ± 10%) were considered inconclusive.

The diagnostic performance of the IBMP chimeric antigens was assessed both individually and using serial and parallel testing approaches. Performance metrics, including sensitivity, specificity, accuracy, likelihood ratios, and diagnostic odds ratio (DOR), were calculated using a two‐by‐two contingency table approach. Overall accuracy was measured by the area under the curve (AUC) and classified as low (0.51–0.61), moderate (0.62–0.81), elevated (0.82–0.99), or outstanding (1.0) [19]. Imprecision was evaluated using Cohen's *Kappa* coefficient, with agreement categorised as poor (*κ* < 0.00), slight (0.00–0.20), fair (0.21–0.40), moderate (0.41–0.60), substantial (0.61–0.80), and almost perfect (0.81–1.00) [20].

**FIGURE 1:**
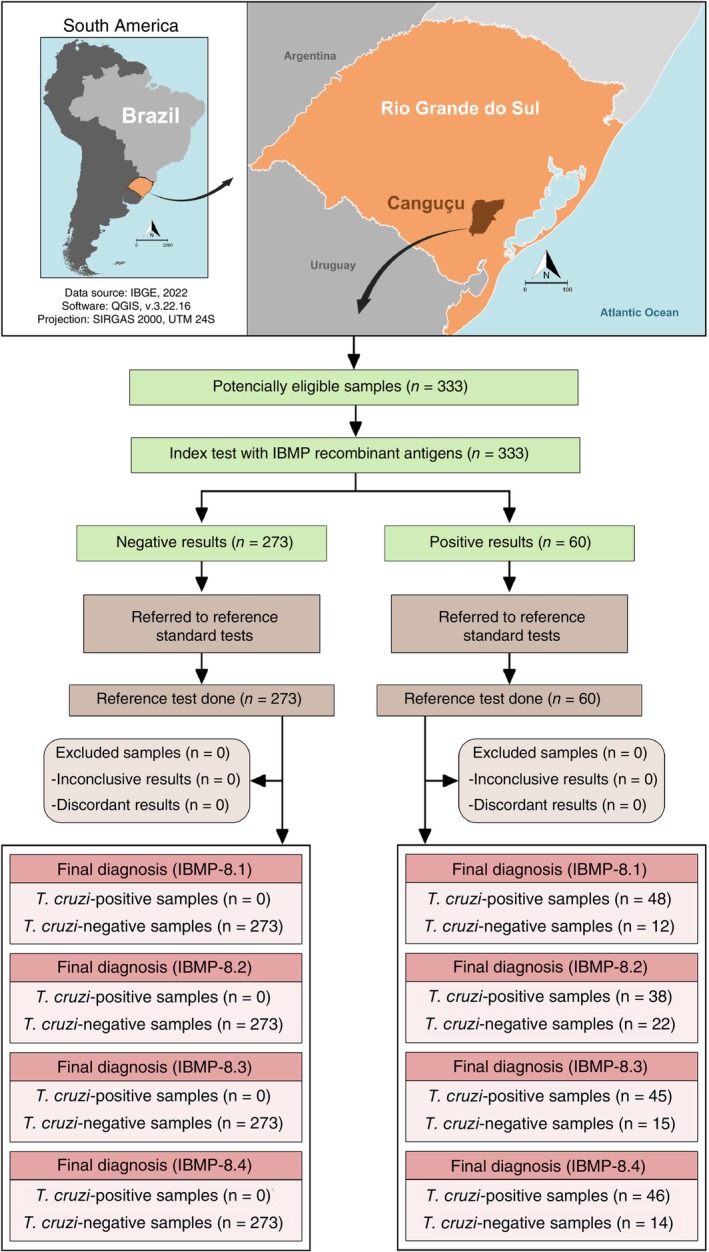
Flowchart depicting the study design in accordance with the Standards for Reporting of Diagnostic Accuracy Studies (STARD) guidelines. Public domain digital maps were obtained from the Brazilian Institute of Geography and Statistics (IBGE) cartographic database in shapefile format (.shp), which was subsequently reformatted and analysed using QGIS version 3.10 (Geographic Information System, Open‐Source Geospatial Foundation Project. http://qgis.osgeo.org).

A checklist (Table S1) and flowchart (Figure 1) were included following the Standards for Reporting Diagnostic Accuracy Studies (STARD) guidelines [21].

## 3 Results

A total of 333 serum samples were analysed in this study. The study population had a median age of 61 years (IQR: 52–70), with a women‐to‐men ratio of 1.68:1. The median age among women was 60 years (IQR: 51–69), which was significantly lower than that of men (63 years; IQR: 55–71) (*p* = 0.043).

The overall prevalence of anti‐ *T. cruzi* antibodies was 18% (60/333), with a significantly higher prevalence in men (25.8%; 32/124) compared to women (13.4%; 28/209) (*p* = 0.019). Among the 60 *T. cruzi* ‐positive samples, 54 tested positive in both the Gold ELISA Chagas and Biolisa Chagas Recombinante assays. The remaining six samples, which were negative in the Gold ELISA Chagas, tested positive in the Biolisa Chagas Recombinante assay. All 60 samples were confirmed as positive using the Chagatest ELISA Recombinante v.4.0.

**FIGURE 2:**
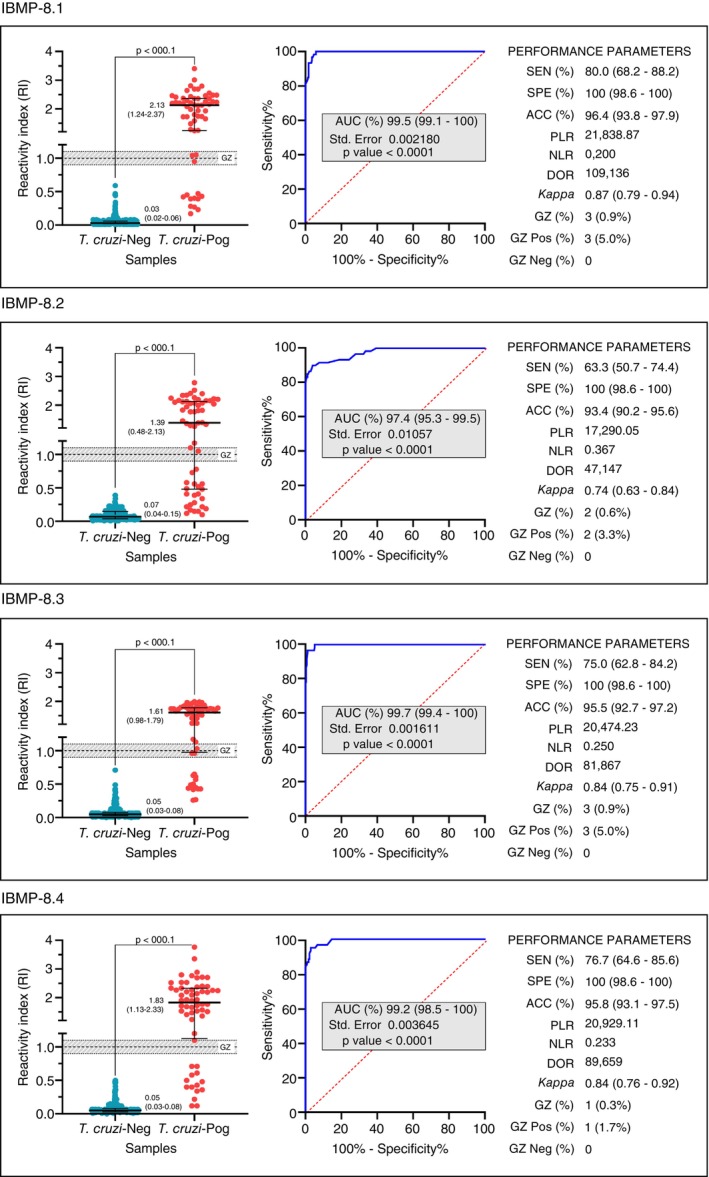
Reactivity index (RI) and diagnostic performance parameters for *Trypanosoma cruzi*‐negative ( *T. cruzi* ‐Neg; *n* = 273) and *Trypanosoma cruzi*‐positive ( *T. cruzi* ‐Pos; *n* = 60) serum samples. The cut‐off value is set at RI = 1.0, with the shaded area indicating the grey zone (RI = 1.0 ± 0.10). Horizontal lines and numerical values denote the interquartile range (IQR) for each group. AUC, Area Under Curve; Sen, Sensitivity; Spe, Specificity; Acc, Accuracy; PLR, Positive Likelihood Ratio; NLR, Negative Likelihood Ratio; DOR, Diagnostic Odds Ratio; GZ, Grey Zone.

The assay performance parameters and reactivity index (RI) distributions for the IBMP chimeric antigens are presented in Figure 2 (individual data points are available in Table S2). ROC curves were generated using 273 *T. cruzi* ‐negative and 60 *T. cruzi* ‐positive samples assayed by ELISA. The AUC values were 99.5% (95% CI: 99.1%–100%) for IBMP‐8.1, 97.4% (95% CI: 95.3%–99.5%) for IBMP‐8.2, 99.7% (95% CI: 99.4%–100%) for IBMP‐8.3, and 99.2% (95% CI: 98.5%–100%) for IBMP‐8.4. No statistically significant differences were observed between AUC values. These findings indicate that the IBMP chimeric antigens demonstrated strong discriminatory power and high diagnostic performance.

For *T. cruzi* ‐positive samples, IBMP‐8.1 exhibited the highest RI values, whereas IBMP‐8.2 showed the lowest RI distribution. However, no statistically significant differences were observed among the RI values of the IBMP antigens. Among *T. cruzi* ‐negative samples, IBMP‐8.1 yielded the lowest RI value, followed by IBMP‐8.3 and IBMP‐8.4. Although IBMP‐8.1 produced the highest RI overall, no significant differences were detected among the antigens.

IBMP‐8.1 demonstrated the highest sensitivity (80.0%), followed by IBMP‐8.4 (76.7%), IBMP‐8.3 (75.0%) and IBMP‐8.2 (63.3%), though these differences were not statistically significant. All four IBMP antigens achieved maximum specificity (100%) (Figure 2). Among the chimeric antigens, IBMP‐8.1 exhibited the highest accuracy (96.4%), whereas IBMP‐8.3 and IBMP‐8.4 showed similar values (~95%). IBMP‐8.2 had the lowest accuracy (93.4%), though no statistically significant differences were observed. Diagnostic odds ratio (DOR) analysis revealed high values for all antigens: 109,136 for IBMP‐8.1, 47,147 for IBMP‐8.2, 81,867 for IBMP‐8.3, and 89,659 for IBMP‐8.4. Among the chimeric antigens tested, IBMP‐8.1 and IBMP‐8.4 exhibited the best overall performance, as indicated by the ROC analysis parameters, particularly their exceptionally high DOR values.

By adopting RI values of 1.0 ± 0.10 as the grey zone (inconclusive interval), we observed that all *T. cruzi* ‐negative samples fell within the conclusive range when tested with all antigens. Among *T. cruzi* ‐positive samples, the number of samples in the grey zone was 1 (1.7%) for IBMP‐8.4, 2 (3.3%) for IBMP‐8.2, and 3 (5%) for IBMP‐8.1 and IBMP‐8.3. The overall proportion of samples in the grey zone was 0.3% for IBMP‐8.4, 0.6% for IBMP‐8.2, and 0.9% for IBMP‐8.1 or IBMP‐8.3.

To minimise diagnostic uncertainty, serial and parallel testing strategies were evaluated using IBMP‐ELISA results (Table 1). These approaches combined the results of two diagnostic tests to optimise performance. Sensitivity consistently increased when ELISA results were analysed in parallel, compared to individual chimeric antigen tests or serial testing. Any combination of IBMP proteins yielded higher sensitivity than individual antigens, with IBMP‐8.1 + IBMP‐8.3 and IBMP‐8.1 + IBMP‐8.4 exhibiting the highest combined sensitivity (*≥* 95%). Regarding specificity, all antigens, either individually or in any combination, achieved 100% specificity under both serial and parallel testing strategies. Accuracy exceeded 99% when IBMP‐8.1 + IBMP‐8.3 or IBMP‐8.1+ IBMP‐8.4 were combined. Although diagnostic accuracy under serial testing was generally lower than individual test performance, parallel testing consistently improved accuracy compared to both individual tests and serial analysis.

**TABLE 1:** Diagnostic performance of IBMP‐ELISA assays evaluated individually and in various IBMP antigen combinations. Additional analyses were conducted using serial and parallel testing approaches.

IBMP antigen	Type	Sen (95% CI)	Spe (95% CI)	Acc (95% CI)
8.1	Individual	80.0 (68.2–88.0)	100 (98.3–100)	96.4 (93.8–97.9)
8.2	Individual	63.3 (50.7–74.4)	100 (98.3–100)	93.4 (90.2–95.6)
8.3	Individual	75.0 (62.8–84.2)	100 (98.3–100)	95.5 (92.7–97.2)
8.4	Individual	76.7 (64.6–85.6)	100 (98.3–100)	95.8 (93.1–97.5)
8.1 + 8.2	Series	50.7 (34.6–65.6)	100 (100–100)	91.1 (88.2–93.8)
	Parallel	92.7 (84.3–97.0)	100 (97.2–100)	98.7 (94.9–99.5)
8.1 + 8.3	Series	60.0 (42.8–74.2)	100 (100–100)	92.8 (89.7–95.4)
	Parallel	95.0 (88.2–98.1)	100 (97.2–100)	99.1 (95.6–99.7)
8.1 + 8.4	Series	61.3 (44.0–75.4)	100 (100–100)	93.0 (89.9–95.6)
	Parallel	95.3 (88.7–98.3)	100 (97.2–100)	99.2 (95.7–99.7)
8.2 + 8.3	Series	47.5 (31.8–62.6)	100 (100–100)	90.5 (87.7–93.3)
	Parallel	90.8 (81.6–96.0)	100 (97.2–100)	98.3 (94.4–99.3)
8.2 + 8.4	Series	48.5 (32.7–63.6)	100 (100–100)	90.7 (87.9–93.4)
	Parallel	91.4 (82.5–96.3)	100 (97.2–100)	98.5 (94.6–99.3)
8.3 + 8.4	Series	57.5 (40.5–72.0)	100 (100–100)	92.3 (89.3–95.0)
	Parallel	94.2 (86.8–97.7)	100 (97.2–100)	98.9 (95.4–99.6)

Abbreviations: Acc, Accuracy; CI, Confidence interval; Sen, Sensitivity; Spe, Specificity.

## 4 Discussion

This study evaluated the diagnostic performance of four IBMP chimeric recombinant *T. cruzi* antigens for detecting anti‐ *T. cruzi* antibodies in an endemic region of Rio Grande do Sul, southern Brazil. The findings demonstrate that all tested IBMP antigens exhibited high specificity (100%), whereas IBMP‐8.1, IBMP‐8.3 and IBMP‐8.4 achieved sensitivities exceeding 75%. These results reinforce the robustness of these antigens for the serological diagnosis of chronic CD and align with previous studies that have highlighted their efficacy across diverse epidemiological settings, regardless of regional differences in *T. cruzi* strain diversity and host immune response variability [13, 14, 15, 16].

The sensitivity values reported in this study were lower than those observed in previous studies involving IBMP antigens [13, 14, 15, 22, 23]. Among the evaluated antigens, IBMP‐8.1 exhibited the highest sensitivity, followed by IBMP‐8.4 and IBMP‐8.3, whereas IBMP‐8.2 demonstrated the lowest sensitivity. In contrast, previous studies using samples from other geographical regions reported the highest sensitivity for IBMP‐8.4, likely due to its broader diversity of antigenic epitopes.

In other endemic Brazilian states, such as Bahia, Minas Gerais, Pernambuco, and Goiás, IBMP‐8.4 exhibited the highest sensitivity among all tested antigens, with values ranging from 98.5% to 100% [13]. Similarly, high sensitivity values for IBMP‐8.4 were observed in studies involving samples from other endemic South American countries (Argentina, Bolivia and Paraguay) [15], as well as Spain [14]. Notably, higher sensitivity values were also reported for the other three IBMP antigens in these studies. Interestingly, in Paraná, a state also located in southern Brazil, IBMP‐8.1 and IBMP‐8.2 achieved sensitivities of 97.2%, whereas IBMP‐8.3 and IBMP‐8.4 reached 100% [13]. This suggests that geographical variation may influence the diagnostic performance of these antigens, possibly due to differences in *T. cruzi* strain distribution and host immune responses across regions.

Conversely, all IBMP antigens achieved 100% specificity. This high specificity aligns with previous evaluations of IBMP chimeric antigens, which have demonstrated minimal cross‐reactivity with several infectious diseases [13, 14], including other trypanosomatids, particularly *Leishmania* spp. [24, 25]. A key advantage of chimeric antigens over conventional *T. cruzi* lysates is their ability to exclude cross‐reactive epitopes that may lead to false‐positive results, particularly in endemic regions where multiple trypanosomatid infections coexist. Similar findings have been reported in other Brazilian states [13], endemic South American countries [15] and Spain [14]. These high specificity values are critical for minimising false‐positive results, a major concern in CD serodiagnosis, especially in non‐endemic areas where cross‐reactivity with *Leishmania* spp. and other parasitic infections can compromise test performance.

The diagnostic odds ratio (DOR) values, a key indicator of test performance, were exceptionally high for IBMP‐8.1 (109,136) and IBMP‐8.4 (89,659), confirming their superior discriminatory power. IBMP‐8.3 and IBMP‐8.2 also exhibited high DOR values (81,867 and 47,147, respectively), reinforcing their utility in clinical and epidemiological applications. These findings are consistent with previous evaluations of IBMP chimeric antigens in both endemic and non‐endemic regions, where similar patterns have been reported [13, 14, 15].

One of the critical challenges in CD serodiagnosis is the presence of inconclusive results, often referred to as the grey zone. By adopting a RI threshold of 1.0 ± 0.10 as the inconclusive interval, we found that all *T. cruzi* ‐negative samples fell within the conclusive range. However, a small proportion of *T. cruzi* ‐positive samples fell into the grey zone, with IBMP‐8.1 and IBMP‐8.3 each having three (5%) samples, IBMP‐8.2 having two (3.3%), and IBMP‐8.4 having only one (1.7%). These rates are notably lower than those observed with traditional recombinant antigens, further demonstrating the improved diagnostic reliability of IBMP chimeric proteins [5, 22, 24].

To minimise diagnostic uncertainty, we also assessed serial and parallel testing strategies using IBMP‐ELISA results. As expected, parallel testing significantly increased sensitivity compared to individual chimeric antigen tests or serial testing, with IBMP‐8.1 + IBMP‐8.3 and IBMP‐8.1 + IBMP‐8.4 achieving the highest combined sensitivity (≥ 95%). In contrast, serial testing resulted in reduced sensitivity while maintaining 100% specificity. These findings suggest that IBMP chimeric antigens can be effectively combined to optimise diagnostic performance, providing flexibility for different clinical and epidemiological scenarios. Although individual IBMP antigens have achieved high sensitivity (> 95%) in previous studies, our findings suggest that, in Rio Grande do Sul, pairing antigens was necessary to reach similarly high sensitivity. This may indicate that local *T. cruzi* strain variability influences antigen performance, but it is necessary to increase the sampling to confirm this hypothesis.

Despite the high performance of IBMP chimeric antigens, some limitations must be acknowledged. Although the sample size was sufficient for robust statistical analysis, larger multicentric studies across different endemic and non‐endemic regions in Rio Grande do Sul would further validate the generalisability of our findings. Additionally, while our results suggest that IBMP chimeric proteins can effectively address regional variability in *T. cruzi* antigenic diversity, further investigations into their performance in field‐based rapid diagnostic tests (RDTs) using IBMP antigens (TR Chagas Bio‐Manguinhos) are warranted.

## 5 Conclusion

In summary, this study provides the first confirmation of the high diagnostic performance of IBMP chimeric *T. cruzi* antigens for detecting anti‐ *T. cruzi* antibodies in the population of Canguçu, an endemic region of southern Brazil. IBMP‐8.1 and IBMP‐8.4 exhibited the best overall performance. The low proportion of inconclusive results and the improved sensitivity achieved through parallel testing highlight the potential of IBMP chimeric proteins as reliable alternatives to conventional serological assays. These findings reinforce the applicability of IBMP antigens for both routine diagnostics and large‐scale epidemiological surveillance, contributing to improved case detection and control efforts for CD. Future studies should prioritise expanding their validation across diverse geographic regions and exploring their integration into rapid diagnostic platforms to enhance accessibility and field applicability.

## Conflicts of Interest

The authors declare no conflicts of interest.

## Supporting information


**Table S1:** STARD checklist. Standards for the Reporting of Diagnostic Accuracy Studies (STARD) checklist for reporting studies of diagnostic accuracy.

**Table S2:** Reactivity Index for diagnostic performance assessment.

## Data Availability

The original contributions presented in the study are included in the article/Supporting Informations; further inquiries can be directed to the corresponding author.
